# Fevers

**Published:** 1904-08-06

**Authors:** 


### Progress in Medicine and Surgery.

### FEVERS.

Typhoid Fever.—Sir William Broadbent,[1] in a few remarks introductory to a study of typhoid fever, mentions the advisability of treating the patient and the disease and not merely the temperature. Influenza apart, he says, the old rule holds good that fever steadily increasing for a week without local inflammation may, with few exceptions, be set down as typhoid. Much has recently been written on the ætiology and prevention of this disease in various quarters of the globe. F. M. Sandwith[2] has studied its incidence in Egypt. He challenges the theory that adult Egyptians rarely suffer from the disease because they have acquired immunity by passing through an attack in childhood. In the post-mortem examination of several hundred children under five years of age, exposed during life to the worst sanitary conditions, evidence of typhoid fever was not found in a single case. At the Kasr-el-Ainy Hospital Sandwith only met with six cases in 12 years among a total of 8,752 patients. Other clinical and post-mortem records point to a similar freedom from this disease among the native Egyptian population, at least in rural districts, in spite of a marked absence of sanitary precautions. Egyptians appear to become more liable in proportion as they adopt the habits of and live with Europeans, as when they congregate in Cairo and other large towns or form armies officered by Europeans. An analysis of 87 cases among private patients, chiefly English, in Cairo, shows that young women are more susceptible than young men, and that young adults are most susceptible. Eighteen months and 65 years were the extremes of age noted. Winter seems the favourite season for the development of this fever. Water appears to play the largest part in carrying infection, but milk, ice cream, uncooked vegetables, flies, and dust are additional ætiological factors. The last two act especially during campaigning. Of the 87 private cases 8 per cent. died, all except one being English ladies. Constipation was marked in 47 per cent., and all these cases recovered. Enteric fever in India is dealt with by A. Duncan,[3] who passes in review the various now exploded theories as to its nature held by different Indian medical officers in the past. Contrary to what appears to be the case in Egypt, typhoid fever is common among the natives in India, and thus the country presents an ever present source of infection for the British soldier, since the soil must be largely impregnated with the bacillus of Eberth. The soldier arrives at an age when he is most susceptible to typhoid, and the supply of susceptible material is kept up by the short service system and the consequent frequent changes of men. If men under 25 years of age were excluded from Indian service it is thought the enteric fever returns would be greatly reduced. The avenues of infection for the soldier are many—in the drinks and sweetmeats he obtains in the bazaars, in the contaminated water or milk he may drink outside or inside the barracks, and in the liability to infection by means of dust and flies where the ground is frequently soiled with infected urine and fæces by native patients. Classical temperature charts are uncommon in native and European cases. Diarrhœa also is less common than in this country. Cardiac failure is a frequent cause of death among Europeans. The serum test is valuable in diagnosis, though in the tropics the formation of "false clumps" may be a source of error. In India a virulent culture of dilution 1 in 10 should be used, and true clumps in which the bacilli cannot all be focussed at once, should be formed in 15 or at the outside 20 minutes. Opportunities for infection being so abundant, Professor Wright's method of protective inoculation, which it is conceded by many is followed by a decided decrease in liability, offers most hope as a prophylactic measure. In China[4] Cantlie finds that Europeans are liable to typhoid fever—very liable in certain parts, *e.g.* in Shanghai, where the disease is reported to be at least ten times as prevalent as in England. The Chinaman, though not immune, appears to be very little liable to the complaint. Possibly, however, he is more vulnerable than the imperfect statistics available show, since the Chinese when very ill prefer native treatment at home, having but a poor opinion of Western medicine, although rather more respect is paid to Western surgery. The Chinaman's sanitary arrangements and his water supply are such as to lend much encouragement to the spread of typhoid fever, but he is protected by his culinary habits. He cooks all his vegetables, drinks tea universally instead of water, and instead of milk takes "congee water," water in which rice has been boiled. Hand-fed infants are also brought up on this, and appear to thrive thereon. A mild type of the disease is prevalent. Nevertheless the case mortality among the Chinese is very high, 57·4 per cent. as against 12 per cent. among the Europeans. The channels of typhoid infection in London[5] are discussed by George Newman. The case rate of enteric has remained fairly constant in the metropolis during the last 10 years, working out at an average of 0·75 per 1,000 persons living. Although the water supply may be the cause of sporadic cases, and water-borne infection probably does occur in London, there is no evidence to show that this is the commonest channel of infection. A study of recent epidemics brings two ætiological factors into prominence—the first infection by personal contact, the second infection by means of contaminated food, especially shellfish. Details of numerous outbreaks which have occurred in the different metropolitan boroughs during the last ten years, and which have been ascribed to more or less direct personal contact by the respective borough medical officers, are given. Thus outbreaks at Hackney, Kensington, Southwark, Hampstead, Shoreditch, and Lambeth are described, in all of which infection seemed directly to depend on gross neglect of personal cleanliness and precautions. Infection is now admitted to be conveyed by the urine and fæces of the sufferer, but, as Newman points out, hitherto writers on general medicine have concurred in regarding infection by contact as quite exceptional. The possibility of infection by means of contaminated shellfish was suggested in 1880 by Sir Charles Cameron, and many observers since then, notably Dr. Newsholme of Brighton, Sir William Broadbent, and others have reported cases presumably due to this cause. Recent outbreaks, ascribed to the eating of polluted oysters, have occurred at Winchester, Southampton, and Portsmouth. An organism identical with *B. typhosus* has on at least three occasions been isolated from cockles derived from polluted beds. In London outbreaks have occurred in several boroughs among groups of persons who had eaten cockles at Southend or some other seaside resort. Considerable outbreaks due to infected milk supply occurred at Blackheath in 1894 and at Plumstead in 1895. Reviewing once again the subject of enteric fever infection in camps, H. H. Tooth,[6] after touching on the more usually accredited means of dissemination points out that overcrowding is not improbably a contributing factor leading perhaps to infection from one case to another by means of the pulmonary exhalations. Colonel Frith, of the United States Army, has already drawn attention to the risk of "comrade infection" in overcrowded tents. Tooth considers that diarrhœa affords a delicate index of the sanitary state of a force and that it is the "stormy petrel" of enteric fever. The ætiology and prevention of typhoid is dealt with at some length by H. E. Leigh Canney. He believes that although various media such as soil, refuse, clothing, and possibly the air may afford the Eberth-Gaffky bacillus a temporary means of existence, its natural habitat is the human body and it would tend to disappear from the community if its diffusion among human hosts could be checked by the protection of water and liquid food avenues from contamination. The bacillus grows practically exclusively in the body of the typhoid patient, is discharged in the alvine and urinary excretions, and is conveyed again into the mouth of another individual without any intermediate stage of maturation or development. The principal avenue of access is water. Dissemination by the other media previously mentioned may give rise to sporadic cases. Entry through the air passages is, in the opinion of this writer, quite exceptional. The preventive measures necessary consist in a very careful and systematic sterilisation of all infected excreta, the protection of water supplies, filtration of water, sterilisation of suspected water and systematic attempts to quickly destroy the bacilli in the bodies of those recovered from the disease. Dr. Canney suggests that every case of typhoid should be compulsorily nursed by at least two nurses specially trained and certificated for the purpose, and supplied wholly or partly at the public expense. Cases amid surroundings wholly unsuitable for securing efficient nursing should be isolated in hospitals or homes. Urotropine should be administered as routine treatment towards the close of the illness, and no patient should be regarded as fit for mixing freely with the community until the urine has been certified as free from the presence of bacilli and the temperature has been normal for a fortnight. Similar precautions should be taken in the Army. With regard to the water supply, "gathering grounds" should be carefully protected from contamination by excreta, refuse, or sewage; pipes and conduits must be rendered and maintained impervious; wells should be deep, and their upper portions protected by impervious masonry from the possibility of surface contamination; spring water should be conveyed to the surface in impervious tubes. In manufacturing artificial mineral waters, the bottles and corks should be sterilised and the water boiled. All suspected water should be boiled. Water used for subsidiary purposes, for washing vegetables, utensils, etc., must be above suspicion. For armies in the field, Dr. Canney advocates the special system of training men and officers in sanitary science, and the formation of "water sections" and "pioneer sections" for supplying the Army with pure fluids and clean camps previously described in detail.

[1] Practitioner, January. [2] Ibid. [3] Ibid. [4] Ibid. [5] Ibid. [6] Ibid. [7] Ibid.

